# Red Blood Cell Deformability, Vasoactive Mediators, and Adhesion

**DOI:** 10.3389/fphys.2019.01417

**Published:** 2019-11-15

**Authors:** Timothy J. McMahon

**Affiliations:** Durham VA Medical Center, Duke University, Durham, NC, United States

**Keywords:** nitric oxide, ATP, S-nitrosothiols, transfusion — H/A, microcirculation, sickle cell anemia, sepsis, respiratory

## Abstract

Healthy red blood cells (RBCs) deform readily in response to shear stress in the circulation, facilitating their efficient passage through capillaries. RBCs also export vasoactive mediators in response to deformation and other physiological and pathological stimuli. Deoxygenation of RBC hemoglobin leads to the export of vasodilator and antiadhesive S-nitrosothiols (SNOs) and adenosine triphosphate (ATP) in parallel with oxygen transport in the respiratory cycle. Together, these mediated responses to shear stress and oxygen offloading promote the efficient flow of blood cells and in turn optimize oxygen delivery. In diseases including sickle cell anemia and conditions including conventional blood banking, these adaptive functions may be compromised as a result, for example, of limited RBC deformability, impaired mediator formation, or dysfunctional mediator export. Ongoing work, including single cell approaches, is examining relevant mechanisms and remedies in health and disease.

## Rbc-Derived Mediators

### Export of Vasoactive Mediators by the Red Blood Cell (RBC)

The main job of the red blood cell is to load oxygen (O_2_) onto its hemoglobin (Hb) in the lung and deliver that O_2_ to respiring tissues throughout the body, while also facilitating the clearance of CO_2_ from tissues to lungs for exhalation into the environment. RBC Hb has evolved to serve these purposes smartly, for example fine-tuning the bias in O_2_ transport to respond to tissue needs by responding to allosteric effectors of Hb function such as pH and temperature. More recently, Hb has been recognized for its ability to export vascular mediators that regulate O_2_-sensitive blood in order to further optimize O_2_ delivery (Jia et al., 1996; McMahon et al., 2002; Sonveaux et al., 2007). For example, RBCs export a vasodilator S-nitrosothiol (SNO) group - pre-formed on Hb from nitric oxide (NO) – in concert with the offloading of O_2_, endowing the RBC (blood) with the ability to flow preferentially where nutrient demand is greatest (as signaled by tissue hypoxia and transduced by the O_2_ sensor Hb itself). Here we review the export of such vasoactive mediators in the contexts of RBC deformation (which can also trigger mediator export) and RBC adhesive interactions with endothelial cells, which can be governed by such intercellular mediators.

### Mechanisms of ATP Export by RBCs

Like other cells, RBCs depend on ATP for energy, and, lacking mitochondria, they generate this ATP via glycolysis. RBCs can export ATP in response to hypoxia or mechanical stress and lysis of ATP is not required for ATP liberation (Kirby et al., 2015). RBCs do not have the capacity for regulated formation or extrusion of vesicles for extracellular export of molecules, although incidental formation of microparticles and nanoparticles derived from RBCs is well recognized (Bennett-Guerrero et al., 2014). Therefore, the conduit for ATP export is likely to involve a channel, hemichannel, or pore. Evidence now suggests that the conduit for regulated export of ATP in response to some stimuli is the hemichannel pannexin 1 (a membrane protein analogous to innexins, an invertebrate homolog of the connexins that form gap junctions. Unlike pannexin, connexin molecules do not appear to be present in RBCs (Keller et al., 2017). Inhibitors or genetic ablation of pannexin 1 blocks the export of ATP in response to hypoxia and other stimuli, (Montalbetti et al., 2011; Zhu et al., 2011; Leal Denis et al., 2013) but they did not appear to attenuate the release of ATP in response to hypotonic stress in the form of 75 mM potassium gluconate (Keller et al., 2017). However, regulated release of ATP may be difficult to assay reliably in the presence of hemolysis that is promoted by hypotonic conditions (see below). The mechanistic link between RBC deformation and ATP export has not been identified. However, recent work has indicated that the cell-volume response to deformation is mediated by the mechanosensor piezo1 calcium channel (Cahalan et al., 2015), and gain-of-function piezo1 mutation has been demonstrated in the RBCs of patients with xerocytosis (Zarychanski et al., 2012; Badens and Guizouarn, 2016). Whether piezo1 may participate in transducing RBC deformation-induced ATP release is unknown.

### RBC Export of the NO Derivative SNO

**FIGURE 1:**
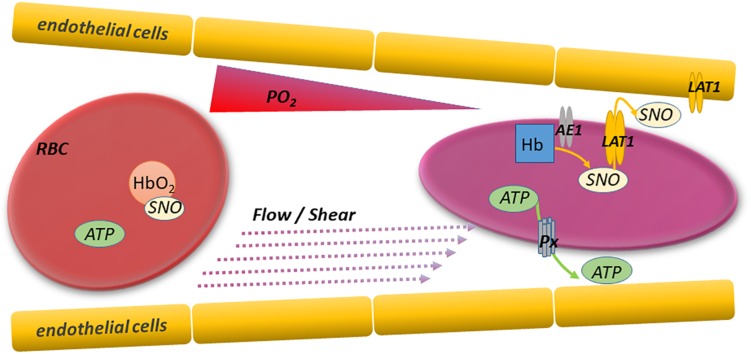
RBC deformation and export of vasoactive and antiadhesive mediators. Red blood cells sense and respond internally and externally to changing conditions as they traverse arterioles and capillaries. Specifically, RBCs deform and offload oxygen in progressively narrower and more hypoxic microvessels perfusing respiring tissues. The resulting RBC deformation and hemoglobin deoxygenation trigger the export of mediators like ATP and SNO that can regulate microvascular tone and prevent intercellular adhesion. This paracrine signaling enables the adaptive and unfettered flow of upstream RBCs as needed, and thereby optimizes O_2_ delivery and CO_2_ clearance. Responsible mediator transporters include pannexin 1 (P×1) for ATP and LAT1 (system L amino acid transporter) for SNO. RBC ATP and SNO can also act via autocrine mechanisms regulating functions including the RBC’s ability to deform in response to shear stress.

Red blood cells exploit allosteric transition cycles in hemoglobin (Hb) to support the production of S-nitrosothiols (SNOs) from nitric oxide (NO) and to drive the export from RBCs of SNOs (Sonveaux et al., 2007). Specifically, deoxygenated or oxygenated human or rodent Hb binds NO avidly at its heme prosthetic groups. Upon transition to the oxygenated state, a redox reaction takes place and a reactive Cys (cysteine) residue in the beta chain (β93 Cys in human Hb) of Hb binds the NO equivalent (which now has NO + or nitrosonium character) (Jia et al., 1996; McMahon et al., 2002). The SNO moiety thus formed in Hb can, as in other proteins, be transferred to a partner protein in a protein transnitrosylation reaction (Foster et al., 2003). For example, Hb can transfer its SNO moiety (Figure 1) to a reactive Cys on RBC membrane resident band 3 (anion exchanger 1) (Pawloski et al., 2001). Deoxygenation drives Hb toward the T state, and allosterically this also promotes the release of SNO from Hb. Coincidentally, Hb-Band 3 interaction is also favored by RBC Hb deoxygenation. The route by which SNO leaves first the cytoplasmic domain of band 3 and then the RBC (Figure 1) can involve the system L amino acid transporter (LAT) on RBC membranes, which efficiently transports CSNO (Dosier et al., 2017). We recently identified a role for LAT1 in the export by RBCs of antiadhesive SNO. Whether deformation of the RBC can promote the export of SNO (as it promotes ATP export) is unknown. Table 1 summarizes key actions and sites of action of exported and intracellular ATP and SNO.

**TABLE 1:** Targets and sites of action of ATP and SNO within and exported by RBCs.

**ATP roles within the RBC**	**Proximal Source**	**Target Cell**	**Physiologic/pathologic Process**	**Method of assay**	**References**
Support metabolic needs	EMP, PPP	RBC	ATP-requiring enzymes such as those for antioxidant defense	HPLC/MS, luciferin/luciferase	Puchulu-Campanella et al., 2013
Provide substrate for regulatory phosphorylation reactions	EMP, PPP	(RBC)	Regulation of solute transport, deformability	Phosphoproteomics, phosphoprotein-specific antibodies	Betz et al., 2009
P2Y receptors: autocrine (negative feedback) G proteins	EMP, PPP	RBC surface	Feedback control of ATP export	ATP flux and receptor pharmacology and genetics	Wang et al., 2005
**Roles of ATP exported by the RBC**					
P2X receptors	EMP, PPP	Leukocytes, endothelial cells	PMN activation; PMN adhesion	Receptor/ligand pharmacology	Dawicki et al., 1995
Unknown	EMP, PPP	Endothelial cells	Inhibition of RBC-EC adhesion	n/a	Zhu et al., 2011
Adenosine receptors after ectonucleotidase activity	ATP from EMP and PPP; ADO from ATPase action	Vascular smooth muscle cells; cardiomyocytes	Vasodilation; modulation of cardiac rhythm	Receptor/ligand pharmacology	Burnstock, 2015
**SNO roles within the RBC**					
GAPDH: regulation of metabolism	Undetermined	RBC	Metabolic regulation	Biotin switch assay	Galli et al., 1998; Foster et al., 2003; Puchulu-Campanella et al., 2013
Spectrin and other cytoskeletal proteins	Undetermined	RBC	Regulation of deformability; role in regulation of adhesion is undetermined	Biotin switch assay	Grau et al., 2013; Riccio et al., 2015
Hemoglobin	Endothelial NO; eNOS in RBCs; NO_2_^–^	RBC	Allosteric coupling of RBC delivery of O_2_ and vasodilator SNO	MPC SNO assays; mouse genetics; SO_2_ gradient clamping	Jia et al., 1996; McMahon et al., 2002; Luchsinger et al., 2005; Cortese-Krott et al., 2012
AE1 (anion exchanger) aka Band 3	Hb-bound SNO	RBC	Membrane-resident stationing of low-mass SNO for export by RBC	Pharmacologic probes	Pawloski et al., 2001
Pannexin 1	Undetermined	RBC	(S)NO regulation of ATP export	Biotin switch assay	Lohman et al., 2012
**Roles of SNO exported by the RBC**					
Soluble guanylate cyclase in vascular smooth muscle	RBC SNO	VSMC	RBC potentiation of hypoxic vasodilation	Pharmacologic inhibitors	McMahon et al., 2005
Platelet activation signaling elements	RBC SNO	PLT	Inhibition of platelet aggregation	Platelet aggregometry	Pawloski et al., 1998
Leukocyte adhesion receptors/ligands	RBC SNO	WBC	Modulation of sickle RBC-driven leukocyte adhesion by NO/SNO	Receptor/ligand antagonists	McMahon et al., 2019
Endothelial adhesion receptors	RBC SNO	EC	Modulation of RBC-EC adhesion	Pharmacologic inhibitors	Dosier et al., 2017

### RBC Auto-Regulation of Exported SNO and ATP

In addition to their direct actions on endothelial cells and RBCs, RBC-derived ATP and NO/SNO can influence one another, an interaction which may serve to fine-tune the loop governing RBC of their (blood) flow. Specifically, ATP exported from RBCs stimulates eNOS in both the RBC itself and in endothelial cells, and the newly synthesized NO can inhibit further ATP export, possibly via S-nitrosylation of the ATP export channel pannexin 1 (Sprague et al., 1996; Olearczyk et al., 2004a, b; Lohman et al., 2012; Ulker et al., 2018). Sprague et al. (1996, 2001) documented impaired ATP release from the RBCs of patients with idiopathic pulmonary arterial hypertension (IPAH, then called primary pulmonary hypertension (PPH). They suggested that the elevated PVR in these patients could be in part secondary to the expected decrease in pulmonary vascular NO synthase activity that results from diminished ATP flux in the pulmonary circulation. ATP can inhibit the activity of Px1, fine-tuning the level of extracellular (exported) ATP (Qiu and Dahl, 2009). This negative feedback effect appears to be mediated by the direct action of ATP on Px1 at extracellular sites bearing homology to P2 × 7 receptors.

## Rbc Deformability and Deformation

### Investigation of RBC Deformability: Evolution of Methodology

The recent advent of more powerful techniques suitable for the study of single cells is accelerating our understanding of how deformability, mediators and adhesivity are linked. Notably, micropipette and filterability methods that allowed early single-cell study of RBC rheology (Canham and Burton, 1968) continue to yield insights. Various methods, differing in strengths and weaknesses, are available to investigators to evaluate RBC deformability. Ektacytometry uses the optical (laser) diffraction pattern to determine the change in length and width of a population of RBCs subjected to varying shear stresses. The commonly measured elongation index (EI) reports the change in length relative to the width as the RBC is stretched progressively by (typically incremental) shear stress. Alternatively, optical tweezer techniques are well suited in the current era of single-cell investigation. Optical force is applied to opposite ends of a visualized cell, and one of the laser beams is moved inward, deforming the cell. The degree of physical displacement of the cell in the perpendicular axis is determined visually. In a variation of the optical tweezers technique, both beams are directed at the cell, well within its boundaries, trapping the cell in place. Then one beam is slowly moved in a direction away from the other, stretching the cell. The degree of cellular narrowing (∼elongation again) is monitored. This approach was used to study the deformability of RBCs from type 2 diabetes mellitus (T2DM) patients with or without diabetic retinopathy in comparison to matched healthy controls (Agrawal et al., 2016). Deformability was modestly but significantly lower in both groups of T2DM patients than in the healthy controls. Notably, Skovburg and coworkers first reported on whole-blood viscosity elevation in diabetes at a time when diabetic RBCs had been reported to have normal viscosity (Skovborg et al., 1966). The single-cell approach and high resolution of this approach allowed the investigators also to document significant differences in the basal RBC length (∼diameter) and to show that deformability (expressed as the elongation index) was inversely related to RBC length. In addition to optical tweezers (or atomic force microscopy, AFM) and standard ektacytometer (e.g., laser-assisted optical red cell rotational analyzer, LORRCA), the ability of RBCs to pass through a filter (“RBC filterability”) of a given size can be assessed as an index of deformability (Oonishi et al., 1997).

### RBC Deformability, Deformation, and RBC Export of ATP in Health

**FIGURE 2:**
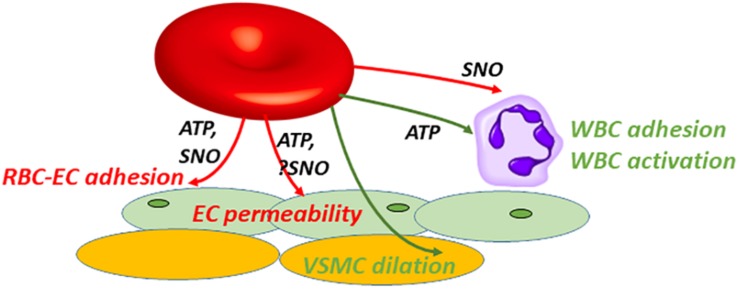
SNO or ATP exported by RBCs can interact with various cell types to produce multiple beneficial or injurious responses in paracrine fashion. Extracellular roles played by RBC-derived SNO or ATP overlap partially. ATP or SNO efflux from vehicle RBCs can be salutary: vasodilation (green); or when preventing (red) adhesion or limiting (red) endothelial (EC) permeability. Note that SNO-induced vasodilation can be endothelium-independent, acting directly on vascular smooth muscle cells (VSMCs). Alternatively these mediators can be injurious, e.g., when acting as a DAMP (damage-associated molecular pattern). ATP can act as a DAMP by promoting (green) leukocyte (WBC) adhesion and activation, driving inflammation in acute lung injury. RBC-derived SNO may oppose WBC adhesion.

Red blood cells (RBCs) must be flexible, and they deform (e.g., elongate) in order to traverse capillaries efficiently. The ability of the discoid healthy RBC (and of RBCs in disease, when the shape may differ from discoid) to deform is a function of the instantaneous properties of the RBC cytoskeleton, the interactions of cytoskeleton with RBC membrane components (proteins and lipids), and the non-cytoskeletal properties of the cytosol, including the cellular hydration state and viscosity (Huisjes et al., 2018). The functional integrity of key cytoskeletal and membrane protein components such as spectrins (alpha and beta) and band 3 (anion exchanger 1; AE1), respectively, are in turn critically dependent on erythrocytic redox state and ATP levels. Cholesterol and other lipids of the RBC membrane play important regulatory functions in RBC deformability as well (Forsyth et al., 2012), in part via effects on fluidity and in turn viscosity. In this review, we discuss consequences of RBC deformation beyond the well-recognized direct implications for blood flow and RBC clearance. Specifically, RBCs sense the mechanical forces leading to deformation and respond by releasing vasoactive mediators that also influence blood flow by acting on the vascular endothelial or smooth muscle cells (Figure 1). For example, ATP exported from deforming RBCs acts via purinergic receptors on endothelial cells to increase endothelial NOS activity. Thus, RBC deformation is an important stimulus for ATP export from RBCs and allows them to dilate arterioles and readily traverse capillaries that are in some cases smaller in diameter than the RBC itself. The degree to which hypoxia and deformation in the microcirculation lower intra-RBC ATP values is unknown, but it is unlikely that there is genuine ATP depletion (with adverse rheologic sequaleae) because extracellular ATP reaches a steady-state value (Montalbetti et al., 2011; Keller et al., 2017) governed in part by the negative feedback mechanisms described at the end of Section I. Figure 2 illustrates the multiple cellular partners potentially influenced by RBC-derived ATP and SNO.

### Impaired RBC Deformability

Impaired RBC deformability (as in RBC senescence, banking, or sickle cell disease) promotes the splenic sequestration of RBCs in narrow splenic sinusoids (Mebius and Kraal, 2005) and subsequently their clearance from circulation (extravascular hemolysis). Poorly deformable RBCs may also impede the flow of blood in other organs. Extravascular hemolytic clearance of RBCs can also be triggered by surface appearance (“externalization”) of normally inward pointing phosphatidylserine (PS) (Boas et al., 1998), a component phospholipid of the membrane lipid bilayer. Other surface receptor or antigen changes can also trigger the splenic clearance of RBCs. Both impaired deformability and PS exposure can result from depression of ATP levels and may therefore represent pathways for the elimination from the circulation of metabolically incompetent RBCs.

## Rbc-Derived Mediators and Endothelial Adhesion

### Single-Cell Examination Suggests a Link Between RBC Adhesion and Deformability

Interestingly, there is marked heterogeneity among sickle (SS) RBCs in terms of their deformability and adhesivity, even among the RBCs of a single individual person with sickle cell disease. Alapan et al. (2014) examined these properties using an innovative and physiologic microfluidic approach coupled with high-resolution imaging in whole blood samples that carry plasma proteins, which may participate in adhesive interactions. In addition to adhesion ligands on endothelial cells and in plasma, the extracellular matrix molecules fibronectin, laminin, and collagens may also bind to RBC surface adhesion molecules. The subset of mature sickle RBCs that are more deformable may paradoxically be the ones most likely to adhere. Papageorgiou et al. (2018) examined the dynamics of the successive establishment of multiple sites of SS RBC adhesion to substrate (fibronectin, mimicking the vessel wall). Poorly deformable cells tended not to establish secondary foci of adhesion. Fewer sites of adhesion translates into weaker RBC-to-wall adhesion overall. The increased SS RBC adhesion measured in hypoxia appears to be secondary, at least in part, to the protrusion of HbS polymer fibers beyond the original cell boundary, increasing the cell surface area potentially available for adhesion. When mature sickle RBCs or sickle reticulocytes were reoxygenated, adhesion persisted, although the cellular protrusions typically receded.

### RBC NO Derivatives, Deformability, and Adhesivity

Riccio et al. (2015) investigated further the link between NO and the deformability and adhesivity of RBCs by examining under what conditions these properties can be corrected in RBCs deficient in Hb-bound NO. RBC banking for later transfusion leads to significant declines in S-nitrosohemoglobin (SNO-Hb) and to increased adhesivity and decreased deformability (Bennett-Guerrero et al., 2007). The NO donor DEA/NO, which releases NO with a half-life of around 15 minutes, effectively promoted restoration of Hb-NO and SNO-Hb, particularly when the incubation was performed anaerobically (Riccio et al., 2015). Anaerobic NO loading may optimize the loading of vacant heme groups within hemoglobin while avoiding oxidative side reactions as in the “methemoglobin (metHb) reaction” where nitrate and metHb are formed when NO and oxyhemoglobin react. As an alternative NO donor, PROLI NON-Oate was also effective, albeit with modestly lower yield relative to that from DEA/NO. Hb-NO yields are dose-dependent, and titration to levels measured in healthy fresh human RBCs led to partial correction of the changes in adhesivity and deformability arising from RBC storage in blood bank conditions. Several recent clinical trials have indicated that clinical outcomes in anemic patients receiving transfusion of RBCs stored for shorter than average periods (e.g., 7–10 days on average rather than 18 days) are not superior (Lacroix et al., 2015; Steiner et al., 2015). However, marked changes take place *early* during RBC storage (for example in pH, glucose, and NO derivatives), and these changes universally present in all banked RBC units may limit the benefit of RBC transfusion. In fact, ill patients with any degree of anemia experience outcomes inferior to patients without anemia but matched for illness severity, (Hebert et al., 1999) but whereas moderate correction of that anemia via RBC transfusion is beneficial, aggressive anemia correction is not beneficial (Hebert et al., 1999; Lacroix et al., 2007; Mazer et al., 2018a, b). This disconnect suggests that blood banked for any amount of time may function inferiorly to native blood, and that those storage lesions present early (arising in the first 7 days) may be important.

### Identifying the Receptors/Ligands Mediating RBC Adhesion Sensitive to Exported Vasoactive ATP

The observation that RBC export of ATP from healthy human RBCs limits basal adhesion of the RBC raised the question of which RBC receptor, and/or which counterreceptor on the endothelial cell is normally inhibited by the continuously released ATP. Using antibodies to candidate adhesion receptors on both the RBC and the endothelial cell, the RBC LW (ICAM-4) and endothelial α_v_β_3_ integrin were identified as responsible (Zhu et al., 2011). The mechanism by which exported ATP acts to limit endothelial adhesion is uncertain, but may involve the stimulation through purinergic receptors of NO production by eNOS in endothelial cells. ECs do produce NO in response to ATP, therefore RBC eNOS is not required (Burnstock, 2008). Beyond receptors and ligands, adhesivity has other determinants. Both RBC and EC surfaces include an external, carbohydrate-based glycocalyx coating. Important roles of the glycocalyx have been described recently, and one such function is to “mask” surface adhesion ligands in order to prevent unwanted adhesion events. The glycocalyx can also participate in transducing endothelial responses to shear stress (McClatchey et al., 2016; Diebel and Liberati, 2019). Additionally, the RBC surface is negatively charged, tending to repel circulating blood cells. The link, if any, between mediators exported by RBCs and these properties remains uncertain.

## Rbc Mediators, Deformation, and Adhesion in Disease States

### Varying Timing of Blood Storage-Induced Changes in RBC Vasoregulatory and Antiadhesive Molecules

Conventional storage of RBCs is carried out using additive solutions designed to mitigate the loss of RBC organic phosphates including ATP and the critical allosteric effector diphosphoglycerate (aka 2,3-DPG, 2,3-BPG or simply “BPG”). The loss of ATP is slowed with the use of the additive solutions in current use, but BPG is nevertheless nearly absent by 14 days of storage. By comparison, shelf life is 35–42 days. Vasoactive NO derivatives in banked RBCs, by contrast, decline more rapidly, with hemoglobin-bound SNO and membrane SNO proteins markedly depressed by 3 h (Bennett-Guerrero et al., 2007). The progressive loss of deformability of stored RBCs takes place over the order of weeks (like that of ATP and DPG) (Bennett-Guerrero et al., 2007). Table 2 summarizes representative changes in RBC deformability in several disease states or conditions, along with the associated changes in export of the mediators ATP and SNO and changes in RBC adhesivity to endothelial cells.

**TABLE 2:** Changes in RBC deformability, adhesivity, and export of the vasoactive mediators ATP and S-nitrosothiols in sickle cell disease, RBC storage or transfusion, and sepsis.

	**Disease or condition**		
	
	**Sickle cell disease**	**RBC transfusion/RBC storage lesion**	**Sepsis**
**RBC function**			
RBC deformability	↓, especially when sickled (Alapan et al., 2014; Huisjes et al., 2018)	↓ progressive over weeks (Bennett-Guerrero et al., 2007; Barshtein et al., 2016)	↓ (Powell et al., 1993; Baskurt et al., 1998)
ATP content ATP export	↓ (Sabina et al., 2009) ↓ (unpublished observations, TJM and M. Telen)	↓ (both) progressive over weeks (Valeri and Hirsch, 1969; Zhu et al., 2011)	↓ (Bateman et al., 2015; Lopez Domowicz et al., 2019) ↓ (Bateman et al., 2015; Lopez Domowicz et al., 2019)
SNO content	↓ (Pawloski et al., 2005)	↓ within hours (Bennett-Guerrero et al., 2007; Reynolds et al., 2007)	**↑** (Jourd’heuil et al., 2000; Crawford et al., 2004; Liu et al., 2004; Doctor et al., 2005)
SNO export or vasoactivity	↓ (Pawloski et al., 2005)	**↓** within hours (Bennett-Guerrero et al., 2007; Reynolds et al., 2007)	**↑** (Crawford et al., 2004)
RBC adhesivity	↑ (Hebbel, 1997; Zennadi et al., 2004)	↑ (Anniss and Sparrow, 2007; Zhu et al., 2011)	↑ by LPS (Eichelbrönner et al., 2003)

### Evolution of RBC “Storage Lesions” in Deformability and Adhesivity

The longstanding question of whether anemic patients transfused with “older” RBC units benefit similarly to those receiving fresher RBC units has recently been addressed in a number of clinical settings. Clinical trials examining this issue were conducted in critically ill adults, cardiac surgery patients, neonates and others, and have used various ranges of storage time in the two study arms. In general, the evidence indicates that transfusion of older RBCs units, at least those in the age ranges employed in these trials, confers no excess harm. However, major changes take place within the first days or even hours of RBC storage. Berezina et al. (2002) demonstrated significant alterations of RBC shape, with the appearance of cells with globular echinocytic or otherwise abnormal shape as early as 5 days. These morphologic changes are accompanied by changes in the deformability of the RBCs and by hemolysis. pH in the RBC unit is below 7.0 in the first days of storage (Hess et al., 2001; Bennett-Guerrero et al., 2007). Given these profound early changes, it is possible that even early changes in banked RBCs may have functional and clinical consequences, a scenario that is compatible with the lack of difference between clinical outcomes in patients given RBC units stored 1–2 weeks vs. units stored for over 2 weeks (Steiner and Stowell, 2009; Fergusson et al., 2012; Lacroix et al., 2015; Steiner et al., 2015; Cooper et al., 2017). Storage also promotes the adhesivity of RBCs, although the precise timing is not well defined (Anniss and Sparrow, 2007; Bennett-Guerrero et al., 2007).

### Post-storage RBC Mediator Restoration

Post-storage RBC mediator restoration can be accomplished when older RBCs are exposed to either renitrosylating (NO/SNO-restoring) or an ATP-repleting (“PIPA,” containing phosphate, inosine, pyruvate, and adenine) solution. Indeed, several lines of evidence link NO derivatives with RBC deformability. Storage-induced changes in RBC deformability have been linked to post-transfusion recipient blood flow (Barshtein et al., 2016). Two structurally dissimilar NOS inhibitors, N^Ω^-nitro-L-arginine methyl ester and S-methylisothiourea, reduced human RBC deformability, effects that were reversible in the subsequent presence of the NO donors sodium nitroprusside (SNP) and diethylenetriamine (DETA)-NON-Oate (Bor-Kucukatay et al., 2003). The rheologic effects of these NOS inhibitors suggested that RBCs may contain NOS, and evidence for an endothelial-type NOS in RBCs was demonstrated several years later (Kleinbongard et al., 2006). Post-storage renitrosylation of RBCs using DEA/NO led to augmented S-nitrosylation of beta-spectrin, a cytoskeletal protein critical for RBC deformation, and attenuated the storage-induced impairment in RBC deformability and the increased adhesivity (Riccio et al., 2015). In mice transfused with RBCs renitrosylated using this strategy, the storage-induced RBC sequestration in the lung and associated impairment in blood oxygenation were ameliorated (Riccio et al., 2015). It is unknown whether augmenting NO or its derivatives can also oppose excessive RBC adhesion in other diseases of the RBC, including sickle cell disease, malarial infection, and diabetes mellitus. Zennadi and colleagues reported that exposing deoxygenated sickle (SS, homozygous) RBCs to NO (followed by reoxygenation, promoting SNO-Hb formation) prevented the proadhesive effect of epinephrine. Hemoglobin-bound SNO is depressed in SS RBCs, and the deficiency tracks with disease severity (Pawloski et al., 2005). The impaired SNO formation may be secondary to a difference in the sickle Hb (HbS) heme’s redox potential relative to that of HbA (the prevalent Hb type in normal adults).

### RBC Mediators and Adhesion in Sickle Cell Disease

Mechanistic investigation of the adhesion of activated sickle RBCs to endothelial cells (ECs), leukocytes, and platelets has yielded important insights into RBC adhesivity in general and therapeutically oriented leads in SCD in particular. Selectins on endothelial and white blood cells, and selectin receptors expressed on RBCs, participate in the “rolling” that characterizes initial contacts of leukocytes with the endothelium. Stronger and more durable intercellular attachment mediated by other ligand-receptor pairs follows but depends on the selectin system for initiation. Selective (anti-P-selectin) and non-specific selectin inhibitors have shown efficacy in phase two clinical trials and in one case a phase three trial. The stronger, secondary vascular adhesion of SS RBCs is mediated by several adhesion receptor/counterreceptor systems, including RBC ICAM-4 (also known as LW) that binds EC α_v_β_3_ integrin, RBC CD44 (hyaluronan receptor), and RBC BCAM/Lu which binds laminin. The reader is referred to excellent reviews specifically addressing mechanisms of RBC adhesion in sickle cell disease for a more comprehensive discussion of relevant adhesion ligands and receptors (Telen, 2005). The relative importance of these receptor/ligand systems varies depending in part on the adhesive stimulus. For example, elevated adhesivity in response to the stress hormone epinephrine acts through ICAM-4 activation with RBC binding to EC α_v_β_3_ integrin. Elements of the intracellular signaling pathways triggered by receptor activation have in some cases been identified and reveal an important node at which RBC ATP may govern adhesivity. Illustrating this is the role of G proteins, adenylate cyclase activation, elevation of cAMP, and the kinases MAPK and MEK in β2-adrenoceptor-mediated proadhesive signaling in response to epinephrine. It is unknown whether therapeutic manipulation of intra-RBC ATP can be leveraged to modulate kinase-dependent adhesion events, and the kinase-ATP link could also be considered in studies of organic phosphate manipulations in RBCs regardless of the primary mechanistic aim (whether targeting O_2_ affinity, ATP preservation for antioxidant defense, or when supplementing RBC ATP in order to boost ATP export capacity).

### Modulation by Sickle RBCs of Endothelial Adhesion of Other Cells

Zennadi et al. (2008) demonstrated that human sickle (SS) RBCs activated by epinephrine and transfused into nude mice promote the adhesion of native peripheral blood mononuclear leukocytes (PBMCs) to endothelium. Adhesion receptors and counterreceptors on each cell type – SS RBC, EC, and WBC – were identified. RBCs can also inhibit the adhesion of other circulating cells: we demonstrated that the coincubation of fresh or 14-day-old healthy (AA) human RBCs with epi-activated SS RBCs attenuated their adhesivity, but that AA RBCs stored 30 days did not attenuate SS RBC adhesivity. The antiadhesive effect could be restored in 30-day-old RBCs by anaerobic NO loading (followed by reoxygenation) that also augmented RBC SNO (including Hb-bound SNO) content (McMahon et al., 2019).

### Adhesion and Deformability of RBCs in Thrombosis

RBCs form a substantial portion of venous thromboses, but their participation has been typically viewed as essentially passive. Disorders of the RBC membrane predispose to thrombosis, (Andrews and Low, 1999) and following the export from RBCs of ATP, endothelial and leukocytic ectonucleotidases can hydrolyze the ATP to proaggregatory ADP, (Hellem et al., 1961) activating platelets and driving clot formation. It is conceivable that not all RBC adhesion is pathogenic. In addition to a homeostatic role in venous thrombosis, endothelial adhesion of RBC precursors may also play an important role in their homing in on supportive niches within the bone marrow microenvironment (Verfaillie, 1998).

### Mechanisms of RBC Clearance and Adhesion Can Overlap

Cell surface ligands and receptors/counterreceptors mediating adhesion events overlap to some extent with those marking cells for clearance, and the RBC is no exception in this regard. Endothelial cells are competent for erythrophagocytosis, and RBC exposure to oxidative stress in response to tert-butyl hydroperoxide induced phosphatidylserine (PS) exposure, promoting subsequent erythrophagocytosis by ECs when opsonizing lactadherin was present (Fens et al., 2012), whether under static or flow conditions. Therefore PS exposure on RBCs, as occurs with RBC aging *in vivo* and in blood banking, promotes both endothelial adhesion and RBC clearance. RBC deformability was depressed in 10 patients with trauma-related sepsis (Powell et al., 1993); the mechanisms are as yet unidentified. The mechanistic study of RBC function in acute and subacute diseases is logistically and technically challenging. We recently described an improved technique for the cryopreservation of human RBCs, resulting in diminished RBC lysis after a cycle of controlled freezing in glycerol followed by thawing and deglycerolization (Rogers et al., 2018), as compared to standard (clinical) RBC cryopreservation. The RBC phenotype with this approach matched closely that of aliquots from the same RBC samples studied before freezing and thawing. Superior cryopreservation is expected to enhance the ability of scientists to examine RBC structure, signaling and function at a time and place of their choosing and thus enable the collection and study of patients with disease states that cannot otherwise be practically investigated. For example, we are now utilizing this approach to collect and examine the molecular and functional basis for sepsis-induced red cell dysfunction.

### Technical Note: Distinguishing Between Experimental Artifact (Hemolysis) and Genuine RBC Export of ATP or SNO in Response to Deformation or Hypoxia

Modeling *in vitro* the hypoxia or deformation encountered by circulating RBCs in order to study the determinants and consequences of the export of vasoactive mediators such as SNO and ATP is challenging. Supraphysiologic levels of mechanical force, resulting either from poorly controlled deformation as the primary stimulus, or from inadvertent mechanical force of gases used to bubble RBC solutions to induce hypoxia, will unquestionably lead to cell rupture and the release of cellular contents (RBC lysis) including ATP. Importantly, the centrifugation used to separate the plasma or supernatant from treated RBCs can itself be sufficient to produce hemolysis, with liberation of ATP (Mancuso et al., 2018). We recommend gentle centrifugation for supernatant separation. When sample manipulation leads to both RBC lysis and ATP liberation, it is not surprising that the extent of hemolysis (% lysis, for example) will correlate numerically with the supernatant ATP concentration (Sikora et al., 2014; Keller et al., 2017). Our approach to ensure scientific rigor and reproducibility in such experiments is to establish conditions in which RBCs can be made hypoxic with minimal mechanical perturbation. This establishes confidence that ATP release in hypoxia (for example) is non-lytic and actually a function of PO_2_ rather than of injurious mechanical force alone (as is sometimes used to “bubble” RBC preparations in order to deoxygenate). Additionally, we advise that in every experiment, the cell-free hemoglobin concentration be measured as an index of RBC lysis. We typically report the extent of hemolysis, and calculate to what extent the measured ATP_EC_ values could theoretically be secondary to the degree of lysis measured (taking into account the initial intra-RBC (ATP) and the hematocrit (Hct)] (Zhu et al., 2011; Rogers et al., 2018). For example, if RBC [ATP] is 100 μM, experiment Hct is 1%, lysis of the RBCs is 5%, and the supernatant [ATP] is 100 nM, then ∼50% of the “ATP_EC_” could be from lysis. In this case, lysis is likely the primary driver of results. However, if under the same conditions lysis is measured at 1% (which alone would yield a supernatant [ATP] of 10 nM), then lysis alone may not account for inter-group differences. It is important to compare the degree of lysis (and the predicted ATP_EC_ resulting from that lysis) between experimental groups.

## Perspective and Conclusion

Deformation of RBCs is necessary for their flow and thus for cellular respiration, and not only promotes the physical passage of RBCs through microvessels, but also leads to the export of vasoactive and antiadhesive mediators, further optimizing flow as a function of need. These paracrine responses are analogous to the metabolically responsive and allosterically regulated export of vasoactive and antiadhesive ATP or SNO in response to RBC Hb deoxygenation. The deformation/mediator export/antiadhesive pathway is disturbed in pathology according to mechanisms that are still being identified. Primary or secondary defects in RBC deformability have the dual effect of directly impairing the ability of the RBC to flow freely through capillaries, and blunting the release of vasoactive mediators necessary for vasoregulation. Meanwhile, approaches to correcting such lesions or exploiting these delivery routes for broader therapeutic gain are being tested, informed by growing recognition of the molecular determinants of RBC mediator formation, release, and activity within the RBC and beyond.

## AUTHOR CONTRIBUTIONS

TM conceived the study and wrote the manuscript.

## Conflict of Interest

The author declares that the research was conducted in the absence of any commercial or financial relationships that could be construed as a potential conflict of interest.

## References

[B1] Agrawal R. Smart T. Nobre-Cardoso J. Richards C. Bhatnagar R. Tufail A. (2016). Assessment of red blood cell deformability in type 2 diabetes mellitus and diabetic retinopathy by dual optical tweezers stretching technique. *Sci. Rep.* 6:15873. 10.1038/srep15873 26976672 PMC4792142

[B2] Alapan Y. Little J. A. Gurkan U. A. (2014). Heterogeneous red blood cell adhesion and deformability in sickle cell disease. *Sci. Rep.* 4:7173. 10.1038/srep07173 25417696 PMC4241514

[B3] Andrews D. A. Low P. S. (1999). Role of red blood cells in thrombosis. *Curr. Opin. Hematol.* 6 76–82. 10088636 10.1097/00062752-199903000-00004

[B4] Anniss A. M. Sparrow R. L. (2007). Variable adhesion of different red blood cell products to activated vascular endothelium under flow conditions. *Am. J. Hematol.* 82 439–445. 10.1002/ajh.20837 17133424

[B5] Badens C. Guizouarn H. (2016). Advances in understanding the pathogenesis of the red cell volume disorders. *Br. J. Haematol.* 174 674–685. 10.1111/bjh.14197 27353637

[B6] Barshtein G. Pries A. R. Goldschmidt N. Zukerman A. Orbach A. Zelig O. (2016). Deformability of transfused red blood cells is a potent determinant of transfusion-induced change in recipient’s blood flow. *Microcirculation* 23 479–486. 10.1111/micc.12296 27406436

[B7] Baskurt O. K. Gelmont D. Meiselman H. J. (1998). Red blood cell deformability in sepsis. *Am. J. Respir. Crit. Care Med.* 157 421–427. 9476853 10.1164/ajrccm.157.2.9611103

[B8] Bateman R. M. Sharpe M. D. Jagger J. E. Ellis C. G. (2015). Sepsis impairs microvascular autoregulation and delays capillary response within hypoxic capillaries. *Crit. Care* 19 389–389. 10.1186/s13054-015-1102-7 26537126 PMC4634189

[B9] Bennett-Guerrero E. Kirby B. S. Zhu H. Herman A. E. Bandarenko N. McMahon T. J. (2014). Randomized study of washing 40- to 42-day-stored red blood cells. *Transfusion* 54 2544–2552. 10.1111/trf.12660 24735194 PMC4194130

[B10] Bennett-Guerrero E. Veldman T. H. Doctor A. Telen M. J. Ortel T. L. Reid T. S. (2007). Evolution of adverse changes in stored RBCs. *Proc. Natl. Acad. Sci. U.S.A.* 104 17063–17068. 10.1073/pnas.0708160104 17940021 PMC2040393

[B11] Berezina T. L. Zaets S. B. Morgan C. Spillert C. R. Kamiyama M. Spolarics Z. (2002). Influence of storage on red blood cell rheological properties. *J. Surg. Res.* 102 6–12. 10.1006/jsre.2001.6306 11792145

[B12] Betz T. Lenz M. Joanny J. F. Sykes C. (2009). ATP-dependent mechanics of red blood cells. *Proc. Natl. Acad. Sci. U.S.A.* 106 15320–15325. 10.1073/pnas.0904614106 19717437 PMC2741249

[B13] Boas F. E. Forman L. Beutler E. (1998). Phosphatidylserine exposure and red cell viability in red cell aging and in hemolytic anemia. *Proc. Natl. Acad. Sci. U.S.A.* 95 3077–3081. 10.1073/pnas.95.6.3077 9501218 PMC19697

[B14] Bor-Kucukatay M. Wenby R. B. Meiselman H. J. Baskurt O. K. (2003). Effects of nitric oxide on red blood cell deformability. *Am. J. Physiol. Heart Circ. Physiol.* 284 H1577–H1584. 12521942 10.1152/ajpheart.00665.2002

[B15] Burnstock G. (2008). Dual control of vascular tone and remodelling by ATP released from nerves and endothelial cells. *Pharmacol. Rep.* 60 12–20. 18276981

[B16] Burnstock G. (2015). Blood cells: an historical account of the roles of purinergic signalling. *Purinergic Signal.* 11 411–434. 10.1007/s11302-015-9462-7 26260710 PMC4648797

[B17] Cahalan S. M. Lukacs V. Ranade S. S. Chien S. Bandell M. Patapoutian A. (2015). Piezo1 links mechanical forces to red blood cell volume. *eLife* 4:e07370. 10.7554/eLife.07370 26001274 PMC4456639

[B18] Canham P. B. Burton A. C. (1968). Distribution of size and shape in populations of normal human red cells. *Circ. Res.* 22 405–422. 10.1161/01.res.22.3.405 5639051

[B19] Cooper D. J. McQuilten Z. K. Nichol A. Ady B. Aubron C. Bailey M. (2017). Age of red cells for transfusion and outcomes in critically III adults. *N. Engl. J. Med.* 377 1858–1867. 10.1056/NEJMoa1707572 28952891

[B20] Cortese-Krott M. M. Rodriguez-Mateos A. Sansone R. Kuhnle G. G. Thasian-Sivarajah S. Krenz T. (2012). Human red blood cells at work: identification and visualization of erythrocytic eNOS activity in health and disease. *Blood* 120 4229–4237. 10.1182/blood-2012-07-442277 23007404

[B21] Crawford J. H. Chacko B. K. Pruitt H. M. Piknova B. Hogg N. Patel R. P. (2004). Transduction of NO-bioactivity by the red blood cell in sepsis: novel mechanisms of vasodilation during acute inflammatory disease. *Blood* 104 1375–1382. 10.1182/blood-2004-03-0880 15150083

[B22] Dawicki D. D. McGowan-Jordan J. Bullard S. Pond S. Rounds S. (1995). Extracellular nucleotides stimulate leukocyte adherence to cultured pulmonary artery endothelial cells. *Am. J. Physiol.* 268 L666–L673. 7733307 10.1152/ajplung.1995.268.4.L666

[B23] Diebel L. N. Liberati D. M. (2019). Red blood cell storage and adhesion to vascular endothelium under normal or stress conditions: an in vitro microfluidic study. *J. Trauma Acute Care Surg.* 86 943–951. 10.1097/TA.0000000000002239 31124891

[B24] Doctor A. Platt R. Sheram M. L. Eischeid A. McMahon T. Maxey T. (2005). Hemoglobin conformation couples erythrocyte S-nitrosothiol content to O2 gradients. *Proc. Natl. Acad. Sci. U.S.A.* 102 5709–5714. 10.1073/pnas.0407490102 15824313 PMC556285

[B25] Dosier L. B. M. Premkumar V. J. Zhu H. Akosman I. Wempe M. F. McMahon T. J. (2017). Antagonists of the system L neutral amino acid transporter (LAT) promote endothelial adhesivity of human red blood cells. *Thromb. Haemost.* 117 1402–1411. 10.1160/TH16-05-0373 28382373 PMC5755361

[B26] Eichelbrönner O. Sibbald W. J. Chin-Yee I. H. (2003). Intermittent flow increases endotoxin-induced adhesion of human erythrocytes to vascular endothelial cells. *Intensive Care Med.* 29 709–714. 10.1007/s00134-003-1698-y 12632262

[B27] Fens M. H. van Wijk R. Andringa G. van Rooijen K. L. Dijstelbloem H. M. Rasmussen J. T. (2012). A role for activated endothelial cells in red blood cell clearance: implications for vasopathology. *Haematologica* 97 500–508. 10.3324/haematol.2011.048694 22102700 PMC3347679

[B28] Fergusson D. A. Hebert P. Hogan D. L. LeBel L. Rouvinez-Bouali N. Smyth J. A. (2012). Effect of fresh red blood cell transfusions on clinical outcomes in premature, very low-birth-weight infants: the ARIPI randomized trial. *JAMA* 308 1443–1451. 23045213 10.1001/2012.jama.11953

[B29] Forsyth A. M. Braunmuller S. Wan J. Franke T. Stone H. A. (2012). The effects of membrane cholesterol and simvastatin on red blood cell deformability and ATP release. *Microvasc. Res.* 83 347–351. 10.1016/j.mvr.2012.02.004 22349292

[B30] Foster M. W. McMahon T. J. Stamler J. S. (2003). S-nitrosylation in health and disease. *Trends Mol. Med.* 9 160–168. 10.1016/s1471-4914(03)00028-5 12727142

[B31] Galli F. Rovidati S. Ghibelli L. Canestrari F. (1998). S-nitrosylation of glyceraldehyde-3-phosphate dehydrogenase decreases the enzyme affinity to the erythrocyte membrane. *Nitric Oxide* 2 17–27. 10.1006/niox.1997.0148 9706739

[B32] Grau M. Pauly S. Ali J. Walpurgis K. Thevis M. Bloch W. (2013). RBC-NOS-dependent S-nitrosylation of cytoskeletal proteins improves RBC deformability. *PLoS One* 8:e56759. 10.1371/journal.pone.0056759 23424675 PMC3570529

[B33] Hebbel R. P. (1997). Perspectives series: cell adhesion in vascular biology. Adhesive interactions of sickle erythrocytes with endothelium. *J. Clin. Investig.* 99 2561–2564. 10.1172/jci119442 9169483 PMC508099

[B34] Hebert P. C. Wells G. Blajchman M. A. Marshall J. Martin C. Pagliarello G. (1999). A multicenter, randomized, controlled clinical trial of transfusion requirements in critical care. Transfusion requirements in critical care investigators, Canadian critical care trials group. *N. Engl. J. Med.* 340 409–417. 9971864 10.1056/NEJM199902113400601

[B35] Hellem A. J. Borchgrevink C. F. Ames S. B. (1961). The role of red cells in haemostasis: the relation between haematocrit, bleeding time and platelet adhesiveness. *Br. J. Haematol.* 7 42–50. 10.1111/j.1365-2141.1961.tb00318.x 13713094

[B36] Hess J. R. Rugg N. Knapp A. D. Gormas J. F. Hill H. R. Oliver C. K. (2001). The role of electrolytes and pH in RBC ASs. *Transfusion* 41 1045–1051. 10.1046/j.1537-2995.2001.41081045.x 11493737

[B37] Huisjes R. Bogdanova A. van Solinge W. W. Schiffelers R. M. Kaestner L. van Wijk R. (2018). Squeezing for life – properties of red blood cell deformability. *Front. Physiol.* 9:656. 10.3389/fphys.2018.00656 29910743 PMC5992676

[B38] Jia L. Bonaventura C. Bonaventura J. Stamler J. S. (1996). S-nitrosohaemoglobin: a dynamic activity of blood involved in vascular control. *Nature* 380 221–226. 10.1038/380221a0 8637569

[B39] Jourd’heuil D. Gray L. Grisham M. B. (2000). S-nitrosothiol formation in blood of lipopolysaccharide-treated rats. *Biochem. Biophys. Res. Commun.* 273 22–26. 10.1006/bbrc.2000.2892 10873557

[B40] Keller A. S. Diederich L. Panknin C. DeLalio L. J. Drake J. C. Sherman R. (2017). Possible roles for ATP release from RBCs exclude the cAMP-mediated Panx1 pathway. *Am. J. Physiol. Cell Physiol.* 313 C593–C603. 10.1152/ajpcell.00178.2017 28855161 PMC5814586

[B41] Kirby B. S. Schwarzbaum P. J. Lazarowski E. R. Dinenno F. A. McMahon T. J. (2015). Liberation of ATP secondary to hemolysis is not mutually exclusive of regulated export. *Blood* 125 1844–1845. 10.1182/blood-2014-11-609610 25766567 PMC4357588

[B42] Kleinbongard P. Schulz R. Rassaf T. Lauer T. Dejam A. Jax T. (2006). Red blood cells express a functional endothelial nitric oxide synthase. *Blood* 107 2943–2951. 10.1182/blood-2005-10-3992 16368881

[B43] Lacroix J. Hebert P. C. Fergusson D. A. Tinmouth A. Cook D. J. Marshall J. C. (2015). Age of transfused blood in critically ill adults. *N. Engl. J. Med.* 372 1410–1418. 10.1056/NEJMoa1500704 25853745

[B44] Lacroix J. Hebert P. C. Hutchison J. S. Hume H. A. Tucci M. Ducruet T. (2007). Transfusion strategies for patients in pediatric intensive care units. *N. Engl. J. Med.* 356 1609–1619. 17442904 10.1056/NEJMoa066240

[B45] Leal Denis M. F. Incicco J. J. Espelt M. V. Verstraeten S. V. Pignataro O. P. Lazarowski E. R. (2013). Kinetics of extracellular ATP in mastoparan 7-activated human erythrocytes. *Biochim. Biophys. Acta* 1830 4692–4707. 10.1016/j.bbagen.2013.05.033 23742824 PMC3999873

[B46] Liu L. Yan Y. Zeng M. Zhang J. Hanes M. A. Ahearn G. (2004). Essential roles of S-nitrosothiols in vascular homeostasis and endotoxic shock. *Cell* 116 617–628. 10.1016/s0092-8674(04)00131-x 14980227

[B47] Lohman A. W. Weaver J. L. Billaud M. Sandilos J. K. Griffiths R. Straub A. C. (2012). S-nitrosylation inhibits pannexin 1 channel function. *J. Biol. Chem.* 287 39602–39612. 10.1074/jbc.M112.397976 23033481 PMC3501028

[B48] Lopez Domowicz D. Herbert J. A. McMahon T. J. (2019). Decreased ATP Export from erythrocytes in murine sepsis. *Am. J. Respir. Crit. Care Med.* 199:A4138.

[B49] Luchsinger B. P. Rich E. N. Yan Y. Williams E. M. Stamler J. S. Singel D. J. (2005). Assessments of the chemistry and vasodilatory activity of nitrite with hemoglobin under physiologically relevant conditions. *J. Inorg. Biochem.* 99 912–921. 10.1016/j.jinorgbio.2004.12.010 15811508

[B50] Mancuso J. E. Jayaraman A. Ristenpart W. D. (2018). Centrifugation-induced release of ATP from red blood cells. *PLoS One* 13:e0203270. 10.1371/journal.pone.0203270 30183749 PMC6124747

[B51] Mazer C. D. Whitlock R. P. Fergusson D. A. Belley-Cote E. Connolly K. Khanykin B. (2018a). Six-month outcomes after restrictive or liberal transfusion for cardiac surgery. *N. Engl. J. Med.* 379 1224–1233. 30146969 10.1056/NEJMoa1808561

[B52] Mazer C. D. Whitlock R. P. Shehata N. (2018b). Restrictive versus liberal transfusion for cardiac surgery. *N. Engl. J. Med.* 379 2576–2577. 10.1056/NEJMc1814414 30586526

[B53] McClatchey P. M. Schafer M. Hunter K. S. Reusch J. E. (2016). The endothelial glycocalyx promotes homogenous blood flow distribution within the microvasculature. *Am. J. Physiol. Heart Circ. Physiol.* 311 H168–H176. 10.1152/ajpheart.00132.2016 27199117 PMC6189750

[B54] McMahon T. J. Ahearn G. S. Moya M. P. Gow A. J. Huang Y. C. Luchsinger B. P. (2005). A nitric oxide processing defect of red blood cells created by hypoxia: deficiency of S-nitrosohemoglobin in pulmonary hypertension. *Proc. Natl. Acad. Sci. U.S.A.* 102 14801–14806. 10.1073/pnas.0506957102 16203976 PMC1253588

[B55] McMahon T. J. Moon R. E. Luchsinger B. P. Carraway M. S. Stone A. E. Stolp B. W. (2002). Nitric oxide in the human respiratory cycle. *Nat. Med.* 8 711–717. 12042776 10.1038/nm718

[B56] McMahon T. J. Shan S. Riccio D. A. Batchvarova M. Zhu H. Telen M. J. (2019). Nitric oxide loading reduces sickle red cell adhesion and vaso-occlusion *In Vivo*. *Blood Adv.* 3 2586–2597. 10.1182/bloodadvances.2019031633 31484636 PMC6737414

[B57] Mebius R. E. Kraal G. (2005). Structure and function of the spleen. *Nat. Rev. Immunol.* 5 606–616. 10.1038/nri1669 16056254

[B58] Montalbetti N. Leal Denis M. F. Pignataro O. P. Kobatake E. Lazarowski E. R. Schwarzbaum P. J. (2011). Homeostasis of extracellular ATP in human erythrocytes. *J. Biol. Chem.* 286 38397–38407. 10.1074/jbc.M111.221713 21921036 PMC3207451

[B59] Olearczyk J. J. Ellsworth M. L. Stephenson A. H. Lonigro A. J. Sprague R. S. (2004a). Nitric oxide inhibits ATP release from erythrocytes. *J. Pharmacol. Exp. Ther.* 309 1079–1084. 10.1124/jpet.103.064709 14766946

[B60] Olearczyk J. J. Stephenson A. H. Lonigro A. J. Sprague R. S. (2004b). NO inhibits signal transduction pathway for ATP release from erythrocytes via its action on heterotrimeric G protein Gi. *Am. J. Physiol. Heart Circ. Physiol.* 287 H748–H754. 15072952 10.1152/ajpheart.00161.2004

[B61] Oonishi T. Sakashita K. Uyesaka N. (1997). Regulation of red blood cell filterability by Ca2+ influx and cAMP-mediated signaling pathways. *Am. J. Physiol.* 273 C1828–C1834. 10.1152/ajpcell.1997.273.6.C1828 9435486

[B62] Papageorgiou D. P. Abidi S. Z. Chang H.-Y. Li X. Kato G. J. Karniadakis G. E. (2018). Simultaneous polymerization and adhesion under hypoxia in sickle cell disease. *Proc. Natl. Acad. Sci. U.S.A.* 115 9473–9478. 10.1073/pnas.1807405115 30190429 PMC6156668

[B63] Pawloski J. R. Hess D. T. Stamler J. S. (2001). Export by red blood cells of nitric oxide bioactivity. *Nature* 409 622–626. 10.1038/35054560 11214321

[B64] Pawloski J. R. Hess D. T. Stamler J. S. (2005). Impaired vasodilation by red blood cells in sickle cell disease. *Proc. Natl. Acad. Sci. U.S.A.* 102 2531–2536. 10.1073/pnas.0409876102 15699345 PMC548996

[B65] Pawloski J. R. Swaminathan R. V. Stamler J. S. (1998). Cell-free and erythrocytic S-nitrosohemoglobin inhibits human platelet aggregation. *Circulation* 97 263–267. 10.1161/01.cir.97.3.263 9462528

[B66] Powell R. J. Machiedo G. W. Rush B. F. Jr. (1993). Decreased red blood cell deformability and impaired oxygen utilization during human sepsis. *AmSurg* 59 65–68. 8480935

[B67] Puchulu-Campanella E. Chu H. Anstee D. J. Galan J. A. Tao W. A. Low P. S. (2013). Identification of the components of a glycolytic enzyme metabolon on the human red blood cell membrane. *J. Biol. Chem.* 288 848–858. 10.1074/jbc.M112.428573 23150667 PMC3543034

[B68] Qiu F. Dahl G. (2009). A permeant regulating its permeation pore: inhibition of pannexin 1 channels by ATP. *Am. J. Physiol. Cell Physiol.* 296 C250–C255. 10.1152/ajpcell.00433.2008 18945939 PMC2643853

[B69] Reynolds J. D. Ahearn G. S. Angelo M. Zhang J. Cobb F. Stamler J. S. (2007). S-nitrosohemoglobin deficiency: a mechanism for loss of physiological activity in banked blood. *Proc. Natl. Acad. Sci. U.S.A.* 104 17058–17062. 10.1073/pnas.0707958104 17940022 PMC2040473

[B70] Riccio D. A. Zhu H. Foster M. W. Huang B. Hofmann C. L. Palmer G. M. (2015). Renitrosylation of banked human red blood cells improves deformability and reduces adhesivity. *Transfusion* 55 2452–2463. 10.1111/trf.13189 26098062 PMC4605860

[B71] Rogers S. C. Dosier L. B. McMahon T. J. Zhu H. Timm D. Zhang H. (2018). Red blood cell phenotype fidelity following glycerol cryopreservation optimized for research purposes. *PLoS One* 13:e0209201. 10.1371/journal.pone.0209201 30576340 PMC6303082

[B72] Sabina R. L. Wandersee N. J. Hillery C. A. (2009). Ca2+-CaM activation of AMP deaminase contributes to adenine nucleotide dysregulation and phosphatidylserine externalization in human sickle erythrocytes. *Br. J. Haematol.* 144 434–445. 10.1111/j.1365-2141.2008.07473.x 19036100 PMC2701153

[B73] Sikora J. Orlov S. N. Furuya K. Grygorczyk R. (2014). Hemolysis is a primary ATP-release mechanism in human erythrocytes. *Blood* 124 2150–2157. 10.1182/blood-2014-05-572024 25097178 PMC4186543

[B74] Skovborg F. Nielsen A. Schlichtkrull J. Ditzel J. (1966). Blood-viscosity in diabetic patients. *Lancet* 287 129–131. 10.1016/s0140-6736(66)91264-5 4158957

[B75] Sonveaux P. Lobysheva I. I. Feron O. McMahon T. J. (2007). Transport and peripheral bioactivities of nitrogen oxides carried by red blood cell hemoglobin: role in oxygen delivery. *Physiology* 22 97–112. 10.1152/physiol.00042.2006 17420301

[B76] Sprague R. S. Ellsworth M. L. Stephenson A. H. Lonigro A. J. (1996). ATP: the red blood cell link to NO and local control of the pulmonary circulation. *Am. J. Physiol.* 271 H2717–H2722. 8997335 10.1152/ajpheart.1996.271.6.H2717

[B77] Sprague R. S. Stephenson A. H. Ellsworth M. L. Keller C. Lonigro A. J. (2001). Impaired release of ATP from red blood cells of humans with primary pulmonary hypertension. *Exp. Biol. Med.* 226 434–439. 10.1177/153537020122600507 11393171

[B78] Steiner M. E. Ness P. M. Assmann S. F. Triulzi D. J. Sloan S. R. Delaney M. (2015). Effects of red-cell storage duration on patients undergoing cardiac surgery. *N. Engl. J. Med.* 372 1419–1429. 10.1056/NEJMoa1414219 25853746 PMC5442442

[B79] Steiner M. E. Stowell C. (2009). Does red blood cell storage affect clinical outcome? When in doubt, do the experiment. *Transfusion* 49 1286–1290. 10.1111/j.1537-2995.2009.02265.x 19602209

[B80] Telen M. J. (2005). Erythrocyte adhesion receptors: blood group antigens and related molecules. *Transfus. Med. Rev.* 19 32–44. 10.1016/j.tmrv.2004.09.006 15830326

[B81] Ulker P. Ozen N. Abdullayeva G. Koksoy S. Yaras N. Basrali F. (2018). Extracellular ATP activates eNOS and increases intracellular NO generation in Red Blood Cells. *Clin. Hemorheol. Microcirc.* 68 89–101. 10.3233/CH-170326 29036803

[B82] Valeri C. R. Hirsch N. M. (1969). Restoration in vivo of erythrocyte adenosine triphosphate, 2,3-diphosphoglycerate, potassium ion, and sodium ion concentrations following the transfusion of acid-citrate-dextrose-stored human red blood cells. *J. Lab. Clin. Med.* 73 722–733. 5779258

[B83] Verfaillie C. M. (1998). Adhesion receptors as regulators of the hematopoietic process. *Blood* 92 2609–2612. 10.1182/blood.v92.8.2609 9763542

[B84] Wang L. Olivecrona G. Gotberg M. Olsson M. L. Winzell M. S. Erlinge D. (2005). ADP acting on P2Y13 receptors is a negative feedback pathway for ATP release from human red blood cells. *Circ. Res.* 96 189–196. 10.1161/01.res.0000153670.07559.e4 15604418

[B85] Zarychanski R. Schulz V. P. Houston B. L. Maksimova Y. Houston D. S. Smith B. (2012). Mutations in the mechanotransduction protein PIEZO1 are associated with hereditary xerocytosis. *Blood* 120 1908–1915. 10.1182/blood-2012-04-422253 22529292 PMC3448561

[B86] Zennadi R. Chien A. Xu K. Batchvarova M. Telen M. J. (2008). Sickle red cells induce adhesion of lymphocytes and monocytes to endothelium. *Blood* 112 3474–3483. 10.1182/blood-2008-01-134346 18664622 PMC2569184

[B87] Zennadi R. Hines P. C. De Castro L. M. Cartron J. P. Parise L. V. Telen M. J. (2004). Epinephrine acts through erythroid signaling pathways to activate sickle cell adhesion to endothelium via LW-alphavbeta3 interactions. *Blood* 104 3774–3781. 10.1182/blood-2004-01-0042 15308566

[B88] Zhu H. Zennadi R. Xu B. X. Eu J. P. Torok J. A. Telen M. J. (2011). Impaired adenosine-5′-triphosphate release from red blood cells promotes their adhesion to endothelial cells: a mechanism of hypoxemia after transfusion. *Crit. Care Med.* 39 2478–2486. 10.1097/CCM.0b013e318225754f 21765360 PMC3196852

